# P-282. Leveraging Machine Learning and Electronic Health Record Data to Identify Patients at Risk of HIV Care Lapses: A Statewide Analysis in Maryland

**DOI:** 10.1093/ofid/ofaf695.503

**Published:** 2026-01-11

**Authors:** Seyed M Shams, Chaitrali-Shiri Kher, Colleen Reilly, Divya Hosangadi, Colleen M Ennett, Elana S Rosenthal, Kristen A Stafford

**Affiliations:** University of Maryland - Institute for Health Computing, North Bethesda, MD; University of Maryland - Institute for Health Computing, North Bethesda, MD; University of Maryland Medical System, Baltimore, Maryland; University of Maryland - Institute for Health Computing, North Bethesda, MD; University of Maryland Medical System, Baltimore, Maryland; Institute for Human Virology IHV, University of Maryland School of Medicine, Washington, District of Columbia; University of Maryland, Baltimore, MD

## Abstract

**Background:**

Retention in HIV care is essential to end the HIV epidemic and improve individual patient outcomes, yet 1/3 of people living with HIV in Maryland are not consistently retained in care. Predictive modeling using electronic health record (EHR) data is a promising strategy to identify patients at risk for lapses in care; however, existing efforts remain limited. Regional analyses using statewide health systems can uniquely inform local strategies by accounting for demographic, clinical, and structural variations. This study aimed to develop predictive models utilizing comprehensive EHR data from the University of Maryland Medical System (UMMS) to identify people living with HIV who are at risk of lapsing in care.

**Figure 1.**
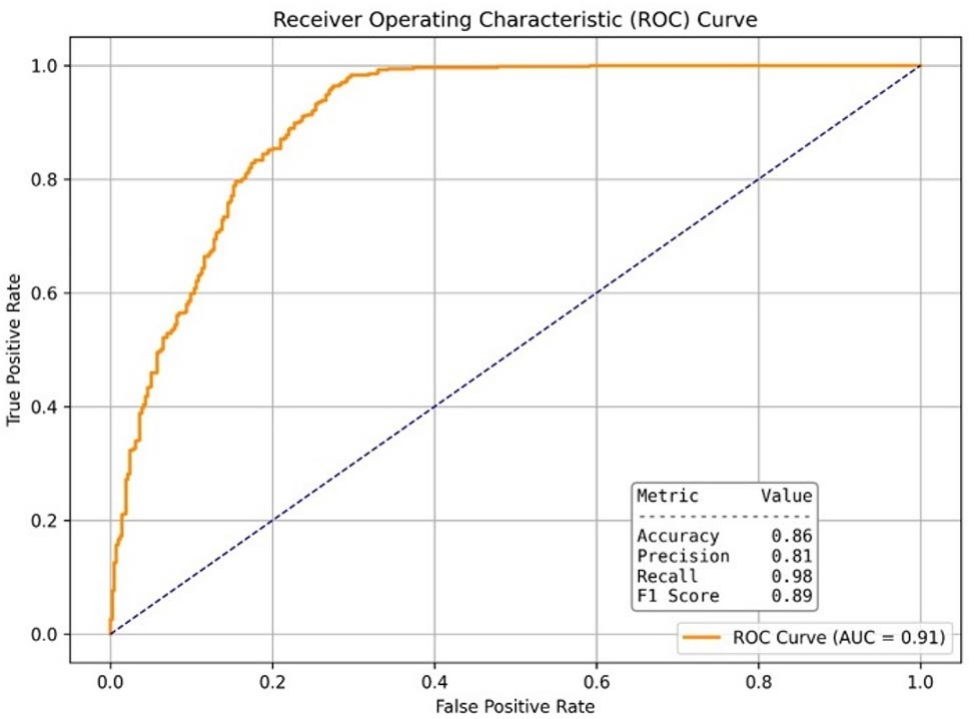
Receiver Operating Characteristic (ROC) Curve demonstrating the performance of the Random Forest model in predicting lapses in HIV care. The model achieved an area under the curve (AUC) of 0.91, with high recall (98%) and precision (81%), indicating strong predictive capability for identifying patients at risk of lapsing in HIV care.

**Figure 2.**
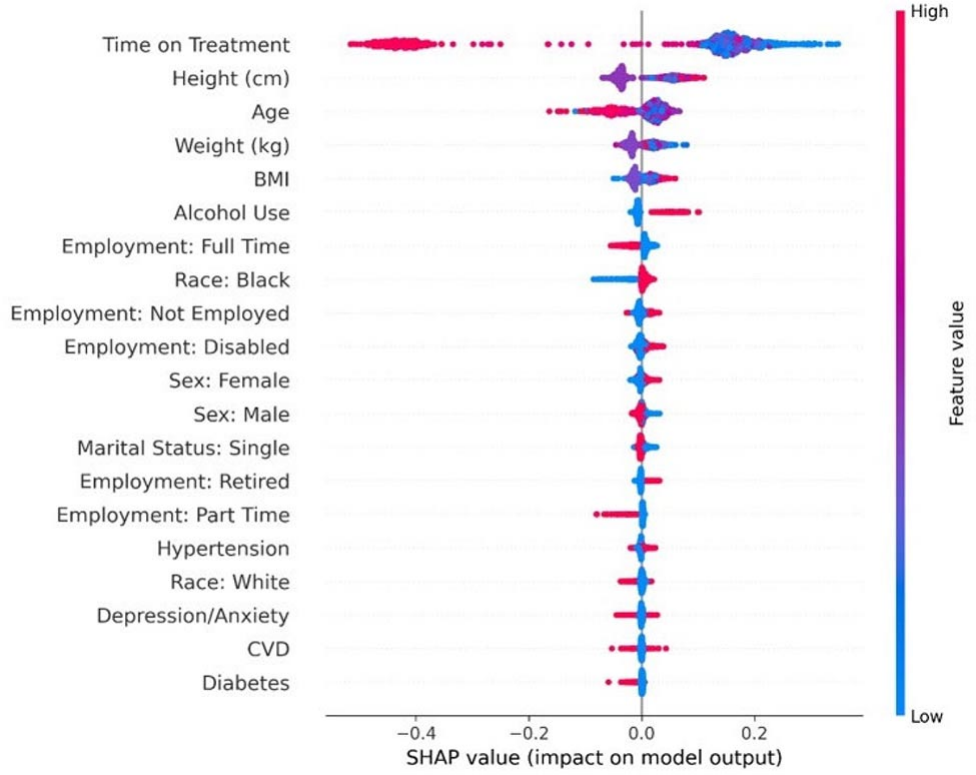
SHAP summary illustrating the impact of key features on the Random Forest model's predictions of HIV care lapses. Each dot represents a patient encounter, with dot colors indicating feature value magnitude (high in red, low in blue).

**Methods:**

Utilizing EHR data from UMMS including HIV-related prescriptions, laboratory tests and clinical visits from adults receiving antiretroviral therapy from 1/2016-6/2024, we identified 8,518 patients with 205,633 encounters. Consolidating multiple encounters within 10 days as one encounter, and including only encounters preceding the last recorded encounter for both lapsed and non-lapsed groups, resulted in 4,735 patients. Of those, 2,667 (56%) had lapses - defined as not having an HIV-related clinical encounter within 12 months. We used a Random Forest classifier with extensive hyperparameter tuning via randomized search cross-validation. Model performance was assessed via the performance matrices and SHAP values for feature importance.

**Results:**

The Random Forest model demonstrated an AUC of 0.91 with 98% recall for lapses, and 81% precision (Figure 1). Feature importance analysis highlighted significant predictors, including time on treatment, age, BMI, alcohol use, employment status, racial demographics, and comorbidities such as hypertension and diabetes (Figure 2).

**Conclusion:**

Predictive modeling using EHR data from a comprehensive statewide health system can effectively identify patients at risk for lapses in HIV care. Clinically actionable predictors, such as treatment duration, demographics, and specific health conditions, provide practical insights that can guide interventions to enhance patient retention and ultimately improve health outcomes for people living with HIV.

**Disclosures:**

All Authors: No reported disclosures

